# Health Insurance Status as a Barrier to Ideal Cardiovascular Health for U.S. Adults: Data from the National Health and Nutrition Examination Survey (NHANES)

**DOI:** 10.1371/journal.pone.0141534

**Published:** 2015-11-04

**Authors:** Michael A. McClurkin, Leah Rae Yingling, Colby Ayers, Rebecca Cooper-McCann, Visakha Suresh, Ann Nothwehr, Debbie S. Barrington, Tiffany M. Powell-Wiley

**Affiliations:** 1 Cardiovascular and Pulmonary Branch, National Heart, Lung, and Blood Institute, National Institutes of Health, Bethesda, Maryland, United States of America; 2 Donald W. Reynolds Cardiovascular Clinical Research Center at the University of Texas Southwestern Medical Center, Dallas, Texas, United States of America; 3 University of Maryland, College Park, Maryland, United States of America; 4 Division of Intramural Research, National Institute on Minority Health and Health Disparities, National Institutes of Health, Bethesda, Maryland, United States of America; University of Louisville, UNITED STATES

## Abstract

**Background:**

Little is known about the association between cardiovascular (CV) health and health insurance status. We hypothesized that U.S. adults without health insurance coverage would have a lower likelihood of ideal cardiovascular health.

**Methods and Results:**

Using National Health and Nutrition Examination Survey (NHANES) data from 2007–2010, we examined the relationship between health insurance status and ideal CV health in U.S. adults aged ≥19 years and <65 (N = 3304). Ideal CV health was defined by the American Heart Association (AHA) as the absence of clinically manifested CV disease and the simultaneous presence of 6–7 “ideal” CV health factors and behaviors. Logistic regression modeling was used to determine the relationship between health insurance status and the odds of ideal CV health. Of the U.S. adult population, 5.4% attained ideal CV health, and 23.5% were without health insurance coverage. Those without health insurance coverage were more likely to be young (p<0.0001), male (p<0.0001), non-white (p<0.0001), with less than a high school degree (p<0.0001), have a poverty-to-income ratio less than 1 (p<0.0001) and unemployed (p<0.0001) compared to those with coverage. Lack of health insurance coverage was associated with a lower likelihood of ideal CV health; however, this relationship was attenuated by socioeconomic status.

**Conclusions:**

U.S. adults without health insurance coverage are less likely to have ideal CV health. Population-based strategies and interventions directed at the community-level may be one way to improve overall CV health and reach this at-risk group.

## Introduction

Cardiovascular disease (CVD) is the leading cause of morbidity and mortality in the United States [1]. Recent data suggest that having a greater number of ideal cardiovascular (CV) health factors and behaviors (i.e. optimal body-mass-index (BMI), blood pressure, fasting blood glucose, and blood lipid levels; adequate physical activity (PA), a healthy diet pattern, and nonsmoking) is inversely associated with lower CVD morbidity and mortality [2–4]. Therefore, population-level interventions that shift CV health behaviors and factors from poor toward ideal are essential.

Unfortunately, ideal CV health is rare in U.S. adults. Less than 5% of U.S. adults achieve ideal CV status, with African Americans disproportionately less likely to have six or seven ideal cardiovascular health factors compared to all other US racial/ethnic groups [1]. Consequently, public health agencies, including the U.S. Department of Health and Human Services through the Healthy People 2020 Guidelines, and organizations such as the American Heart Association (AHA) have outlined initiatives and prioritized strategies to improve CV health and shift CV health factors towards ideal for the U.S. population [5, 6].

Inadequate access to health care services likely poses a challenge to improving the CV health of the U.S. population. Studies have shown that uninsured U.S. adults are less likely to receive medical care, particularly preventive services including routine testing and screening for CVD risk factors [7, 8]. In 2013, 42 million Americans lacked health insurance coverage [9]. Despite expanded and improved access through the Patient Protection and Affordable Care Act in 2014, lack of health insurance coverage remains common among U.S. adults, with gross disparities across racial/ethnic groups and age [10]. Multiple studies have found an association between lack of health insurance and all-cause mortality [11–13]. However, no study to date has looked at the relationship between health insurance coverage and ideal cardiovascular health, a clear precursor to reduced cardiovascular morbidity and mortality [2, 3].

Thus, we sought to understand the relationship between health insurance coverage and CV health in a nationally representative U.S. adult population using National Health and Nutrition Examination Survey (NHANES) data from 2007–2010. We hypothesized that those U.S. adults without health insurance coverage would have a lower likelihood of ideal CV health.

## Methods

### National Health and Nutrition Examination Overview

The National Health and Nutrition Examination Survey (NHANES) is a series of cross-sectional surveys designed by the Centers for Disease Control’s National Center for Health Statistics (NCHS). The survey is conducted using a multistage, stratified sampling design to assess the health and nutritional status of a nationally representative sample of the civilian, non-institutionalized U.S. population. In-home medical history interviews were conducted to gather demographic, socio-economic and health-related information. Participants also underwent a standardized physical examination and completed a 24-hour dietary recall and a dietary behavior questionnaire at mobile examination centers (MEC). The first 24-hour recall for each participant was conducted in-person with English- or Spanish-speaking dietary interviewers. Detailed information regarding NHANES data collection has been previously reported [14]. NHANES data from 2007–2010 were merged for this analysis. Earlier cycles were not included because the physical activity questionnaire changed between the 05–06 and 07–08 cycles.

### Ethics Statement

The study was conducted according to the guidelines in the Declaration of Helsinki and all procedures involving human subjects were approved by the National Center for Health Statistics Institutional Review Board. Written informed consent was obtained from all participants 18 years of age and older.

### Sample Demographics

Age, sex, race and ethnicity, education, poverty-to-income ratio (PIR) and employment categories were defined using self-reported demographic data from 2007–2010 NHANES. Four distinct age groups were included in our analysis: 19–29 years, 30–39 years, 40–49 years, and 50–64 years. Racial and ethnic groups were characterized based on responses to questions about race and Hispanic origin. We included non-Hispanic White, non-Hispanic Black, and Mexican American persons who reported a single racial identity because the group sample sizes were adequate for separate evaluation and estimates [15]. Level of education was categorized as less than high school, completed high school/equivalent, some college, and completed college. The PIR is a ratio of family income to the Health and Human Services (HHS) federal poverty threshold that accounts for inflation and family size. A PIR below 1 indicates that the family income is less than 130% of the poverty threshold, a PIR of 1–3 corresponds to 131%-185% of the poverty threshold, and a PIR of greater than 3 corresponds to income greater than 185% of the poverty threshold. Employment was defined as unemployed, employed (working at a job or business), or employed, but not working (with a job or business, but not at work).

### Cardiovascular Health Factors

Four CV health factors were measured during the physical examination (blood pressure, body mass index, cholesterol, fasting blood glucose), and three CV factors were determined by self-report (smoking status, physical activity, and dietary intake). Three separate blood pressure measurements were taken during the physical exam and averaged to calculate each individual’s blood pressure [16]. Ideal blood pressure was defined as systolic <120 mm Hg and diastolic <80 mm Hg, intermediate was defined as systolic 120–139 mm Hg, diastolic 80–90 mm Hg, or treated to goal, and poor was defined as systolic ≥140 mm Hg or diastolic ≥90 mm Hg. Weight and height were measured during NHANES physical examinations at the MEC. Body mass index (BMI) was calculated as weight in kilograms divided by height in meters squared and rounded to the nearest tenth. Consistent with standard weight ranges of normal, overweight and obese, BMI was categorized as ideal, intermediate and poor. Ideal was defined as BMI 18.5–24.9 kg/m^2^, intermediate was defined as BMI 25–29.9 kg/m^2^, and poor was defined as BMI ≥30 kg/m^2^. Blood samples were collected from NHANES participants during their physical examination. Further procedures for blood processing are described elsewhere [14]. Total blood cholesterol and fasting blood glucose measurements for NHANES participants were determined from their blood sample. Ideal total blood cholesterol was defined as <200 mg/dl untreated, intermediate total blood cholesterol was defined as 200–239 mg/dl or treated to goal, and poor total blood cholesterol was defined as ≥240 mg/dl. Ideal fasting blood glucose was defined as <100 mg/dl with no history of diabetes mellitus. Intermediate fasting blood glucose was defined as 100–125 mg/dl or treated to goal. Poor fasting blood glucose was defined as ≥126 mg/dl.

Ideal smoking status was defined as never smoker, intermediate was defined as former smoker, and poor was defined as current smoker. Ideal physical activity (PA) was defined as ≥150 minutes per week of moderate activity or ≥75 minutes per week of vigorous activity or ≥150 minutes per week of combined moderate and vigorous activity; intermediate was defined as 1–149 minutes per week of moderate activity or 1–74 minutes per week of vigorous activity or 1–149 minutes per week of combined moderate and vigorous activity; and poor was defined as no reported physical activity.

#### Dietary Intake

The dietary portion of NHANES is conducted as a partnership between the U.S. Department of Agriculture (USDA) and HHS to estimate the types and amounts of foods and beverages consumed by respondents during the 24-hour period prior to the interview. Two 24-hour dietary recalls were collected by trained dietary interviewers: an in-person interview on Day 1 at the MEC and a telephone interview collected 3–10 days after Day 1. All participants who had reliable 24-hour recall data from Day 1 were included in our study population. Day 1 of the 24-hour recall data was used because the majority of participants completed Day 1 testing during the MEC examination, and the setting was consistent for all participants.

Each food or beverage reported during the 24-hour recall was coded with a specific food code from the US Department of Agriculture Food and Nutrient Database for Dietary Studies (FNDDS), a database of foods and beverages, nutrient values and weights for portions. NHANES 2007–2008 and NHANES 2009–2010 data were coded with FNDDS 4.1 and FNDDS 5.0, respectively[17, 18]. The USDA’s Food Patterns Equivalents Database (FPED) was used to translate single and multi-ingredient foods into cup, ounce (oz.) or teaspoon equivalents and to convert reported foods into nutritionally meaningful groupings. The database disaggregates reported foods into ingredients and classifies the ingredients into one of 37 predefined groupings (“components”), such as whole grains or dark green vegetables [19, 20]. FPED 07–08 and FPED 09–10 correspond with NHANES 07–08 and NHANES 09–10.

Fruit and vegetable servings were measured in cup equivalents. Fruits included whole, dried, and canned fruit, mixed fruit dishes and fruit juice. Vegetables included dark green vegetables, red and orange vegetables, starchy vegetables, and excluded beans and legumes. The sum of the total fruits and vegetables for each respondent represented total fruit and vegetable intake measured in cups. Fish servings were measured in cup equivalents and included fish high and low in n-3 fatty acids. Fiber-rich whole grains were measured in ounce equivalents and included those whole grains that contain the entire grain kernel ― the bran, germ, and endosperm. Dietary sodium intake in milligrams per day was measured using the Total Nutrient Intake File [21, 22]. Estimated sodium intake does not include salt added at the table. Consistent with other studies [23, 24], sugar-sweetened beverages (SSBs) were defined as any non-diet, non-alcoholic beverage items and beverage concentrates with added sugars. SSBs included soda, fruit drinks, energy and sports drinks, sweetened coffee, sweetened tea, liquid and dry beverage concentrates and sweetened bottled waters, all with added sugars. Those beverages that were sweetened after purchase were not considered a SSB. SSBs were measured in kilocalories per week. Because FPED values for added sugars are measured in teaspoons per day, a standard 16.8 kcal/teaspoon of added sugars was used as a conversion factor for calculating kilocalories from added sugars in SSBs. Total kilocalories was multiplied by seven to represent weekly caloric intake from added sugars in SSBs.

To evaluate the dietary intake of US adults, we computed a healthy diet score, previously described by Lloyd-Jones *et al* [5] and consistent with the current Dietary Guidelines for Americans [25] and AHA recommendations [26, 27]. The healthy diet score sets ideal criteria for five constituents of dietary intake: fruits and vegetables [≥4.5 cups per day], fish [≥ two 3.5-oz servings per week (preferably oily fish)], fiber-rich whole grains [≥ three 1-oz-equivalent servings per day], sodium [<1500 mg per day], and SSBs [≤450 kcal (36 oz.) per week]. Ideal dietary intake was defined as meeting 4 to 5 components of the healthy diet criteria. Intermediate was defined as meeting 2 to 3 components. Poor was defined as meeting 0 to 1 components.

Overall, ideal CV health was defined as meeting ideal across 6–7 CV health factors, intermediate CV health was defined as meeting ideal across 3–5 CV health factors, and poor CV health was defined as meeting ideal across 0–2 ideal CV health factors.

### Study Population

For our analysis, we included data on those US adults aged 19–64 who had a non-missing sample weight and a non-missing questionnaire response to the health insurance coverage question. We excluded those aged 65 and older, as an overwhelming majority of these individuals have health insurance through Medicare. In agreement with previous studies [11, 28, 29], we also excluded individuals covered under the Department of Veterans Affairs/Civilian Health and Medical Program of the Uniformed Services military insurance and non-elderly Medicare due to poor health being a prerequisite for coverage for most individuals under these plans. Of the 3576 individuals with a non-missing insurance status, non-missing sample weight and within the age range of 19–64 years, 272 (7.6%) had incomplete information for one or more of the 7 CV health factors, resulting in a final study population of 3304 NHANES participants. Of the 272 participants excluded, 105 had incomplete BP data, 0 had incomplete blood glucose data, 19 had incomplete cholesterol data, 1 had incomplete PA measurement data, 0 had incomplete dietary data, 124 had incomplete smoking data, and 33 had incomplete BMI data. Some participants had incomplete data for multiple CV factors.

### Statistical Analysis

A multistage, probability sampling design was used to make our cohort representative of the civilian, non-institutionalized U.S. population. The sample-weighted chi-square test was used to compare the percentage of uninsured across socio-demographic categories. An age-adjusted, sample-weighted linear regression model was used to compare the percentage of uninsured across CV health factor categories. Sample-weighted logistic regression models were developed to assess the relationship between health insurance coverage and the likelihood of ideal cardiovascular health. Results from logistic models are shown as unadjusted, adjusted for age, race and sex (Model 1), and adjusted for age, race, sex and socioeconomic status (SES) (Model 2). All analyses were performed using SAS, version 9.2, in 2014–2015.

## Results

The sample-weighted percentages of uninsured U.S. adults for the 2007–2010 NHANES population across socio-demographic categories are shown in **Table 1**. Of the U.S. adult population, 76.5% (n = 2225) were with health insurance coverage, while 23.5% (n = 1079) were without coverage. U.S. adults without health insurance were more likely to be young (p<0.0001), male (p<0.0001), non-white (p<0.0001), have less than a high school level education (p<0.0001), have a PIR less than 1 (p<0.0001) and unemployed (p<0.0001) compared to those with health insurance **(Table 1)**.

**Table 1:** Sample-weighted Baseline Characteristics of 2007–2010 NHANES Population (N = 3304) by Health Insurance Status.

Baseline Characteristics		*N* ^a^	% Uninsured (SE)	*p*-value
**Age**	19–29	681	37.8 (2.3)	< .0001
	30–39	701	24.0 (2.3)	
	40–49	768	20.0 (1.6)	
	50–64	1154	15.3 (1.4)	
**Sex**	Female	1714	20.3 (1.2)	< .0001
	Male	1590	26.7 (1.3)	
**Race**	Non-Hispanic White	1488	16.6 (1.3)	< .0001
	Non-Hispanic Black	647	29.5 (2.1)	
	Mexican American	1169	49.7 (2.1)	
**Education**	<High school	896	52.7 (2.0)	< .0001
	High school/equivalent	775	29.3 (2.5)	
	Some college	932	20.5 (1.5)	
	College graduate	698	7.4 (1.2)	
**Poverty-to-Income Ratio**	<1	672	52.6 (2.8)	< .0001
	1–3	1214	32.9 (1.9)	
	>3	1122	7.3 (1.1)	
**Employment**	Unemployed	1015	30.1 (1.7)	< .0001
	Employed, working^b^	2208	21.4 (1.1)	
	Employed, not working^c^	81	11.9 (3.7)	

SE = Standard Error

^a^ Sample size (*N*) for each group represents total unweighted *N* for individuals in each group.

^b^ Employed, working, includes those working at a job or business

^c^ Employed, not working, includes those with a job or business, but not working

Of U.S. adults included in this analysis, 5.4% (n = 141) had ideal CV health (6–7 ideal CV health factors), 58.3% (n = 1839) had intermediate CV health (3–5 ideal CV health factors), and 36.3% (n = 1324) had poor CV health (0–2 ideal CV health factors). Only 0.02% of the population had ideal status for all 7 CV health factors. Among the individuals with ideal, intermediate, and poor CV health, the age-adjusted percent distribution with standard errors for those without health insurance coverage was 6.3%±3.1% (n = 31), 23.6%±1.1% (n = 650), and 24.9%±1.5% (n = 398), respectively (p < .0001).

**Table 2** displays the sample-weighted, age-adjusted percentage of uninsured U.S. adults for the 2007–2010 NHANES population across ideal, intermediate or poor categories for each of the seven CV health factors and behaviors. With regard to health factors, U.S. adults with poor fasting blood glucose (uncontrolled diabetes), and poor blood pressure levels (uncontrolled hypertension) were more likely to be uninsured. Those with poor status for BMI (BMI ≥ 30 kg/m^2^) and blood cholesterol were less likely to be uninsured. For the health behaviors, those with poor smoking status (current smokers) and with a poor level of physical activity (none reported per week) were also more likely to be uninsured. There was no statistically significant relationship between dietary intake and insurance status.

**Table 2:** Sample-weighted, Age-Adjusted Cardiovascular Health Factors of 2007–2010 NHANES Population (N = 3304) by Health Insurance Status.

Baseline Characteristics		*N* ^d^	% Uninsured (SE)	*p*-value
**Body Mass Index**	Ideal (Normal: 18.5–24.9 kg/m^2^)	853	23.7 (1.8)	< .0001
	Intermediate (Overweight: 25–29.9 kg/m^2^)	1144	23.3 (1.4)	
	Poor (Obese: ≥30 kg/m2)	1307	22.6 (1.7)	
**Dietary Intake** ^e^	Ideal (4–5 Healthy Diet components)	5	42.4 (24.7)	0.0953
	Intermediate (2–3 Healthy Diet components)	564	21.3 (1.8)	
	Poor (0–1 Healthy Diet components)	2735	23.5 (1.0)	
**Cholesterol**	Ideal (<200 mg/dL untreated)	1620	24.7 (1.4)	< .0001
	Intermediate (200–239 mg/dl or treated to goal)	1234	21.4 (1.4)	
	Poor (≥240 mg/dL)	450	22.6 (2.3)	
**Smoking Status**	Ideal (Never smoker)	1820	18.8 (1.2)	< .0001
	Intermediate (Former Smoker)	672	20.5 (1.6)	
	Poor (Current Smoker)	811	36.0 (2.0)	
**Fasting Blood Glucose**	Ideal (<100 mg/dL w/o Hx of DM)	1687	22.2 (1.3)	< .0001
	Intermediate (100–125 mg/dL or treated to goal)	1310	23.4 (1.4)	
	Poor (≥126 mg/dL)	307	30.4 (2.5)	
**Blood Pressure**	Ideal (SBP < 120 mmHg, DBP < 80 mm Hg)	1615	21.7 (1.4)	< .0001
	Intermediate (SBP 120–139 mmHg, DBP 80–90 mmHg)	1295	21.9 (1.6)	
	Poor (SBP ≥ 140 mm Hg or DBP ≥ 90 mm Hg)	394	36.8 (3.0)	
**Physical Activity (minutes/week)**	Ideal (≥150 mod/ ≥75 vig/ ≥150 mod/vig)	2132	22.1 (1.0)	< .0001
	Intermediate (≤149 mod/ ≤74 vig/ ≤149 mod/vig)	444	19.5 (2.5)	
	Poor (None)	728	30.2 (2.2)	

Hx, history; DM, Diabetes Mellitus; SBP, systolic blood pressure; DBP, indicates diastolic blood pressure; mod, moderate; vig, vigorous

^d^ Sample size (*N*) for each group represents total unweighted *N* for individuals in each group.

^e^ Healthy Diet components: Fruits and vegetables: >4.5 cups per day; Fish: >two 3.5-oz servings per week (preferably oily fish); Fiber-rich whole grains (>1.1 g of fiber per 10 g of carbohydrate): > three 1-oz-equivalent servings per day; Sodium: <1500 mg per day; Sugar-sweetened beverages: <450 kcal (36 oz. per week) per week

**Fig 1:**
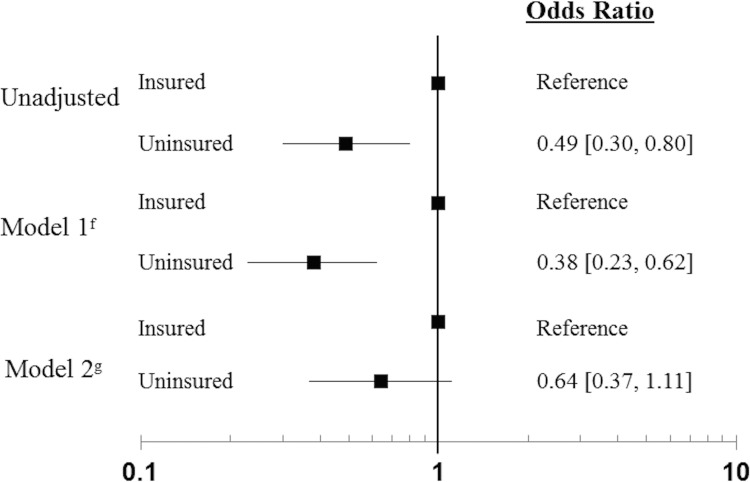
Odds of Ideal Cardiovascular Health of NHANES 2007–2010 Population (N = 3304) by Health Insurance Status. ^f^Model 1: Adjusted for age, sex, race; ^g^Model 2: Adjusted for age, sex, race, SES.

**Fig 1** displays the odds of ideal CV health of the NHANES 2007–2010 adult population by health insurance status. U.S. adults without health insurance were 51% less likely to have ideal CV health compared to U.S. adults with health insurance coverage (OR = 0.49, CI = 0.30–0.80), a relationship that remained significant after adjustment for age, race and sex (OR = 0.38, 95% CI = 0.23–0.62). This relationship no longer remained significant after adjusting for SES (OR = 0.64, 95% CI = 0.37–1.11). For both models 1 and 2, there was neither a sex (p = 0.76 and p = 0.76, respectively) nor race (p = 0.13 and p = 0.26, respectively) interaction. Post hoc analyses revealed that education was the most dominant explanatory SES variable associated with ideal CV health based on c-statistics (data not shown). However, the combined model adjusting for insurance status, education, and income provided a significantly better fit than the models considering individual SES variables only.

## Discussion

In our logistic regression analysis of an ethnically diverse cohort of U.S. adults, we found that those without health insurance coverage were less likely to have ideal cardiovascular health, independent of age, sex, and race. Adjusting for individual-level SES attenuated this relationship. With regard to CV health factors, U.S. adults with poor fasting blood glucose and poor blood pressure levels were more likely to be uninsured. Those with poor status for BMI were less likely to be uninsured. Those with ideal total cholesterol levels were more likely to be uninsured than those with poor total cholesterol levels. With regards to CV health behaviors, those with poor smoking status and with a poor level of physical activity were also more likely to be uninsured. Thus, targeting policy efforts that aim to increase healthcare coverage may aid in improving and sustaining certain individual level CV health factors and behaviors, thus improving the overall CV health of adults in the United States.

This is one of the first studies to assess the relationship between health insurance coverage and prevalence of ideal CV health. Our findings suggest that the lack of health insurance coverage may be a barrier to achieving ideal cardiovascular health for U.S. adults. Consistent with previous work, our findings also suggest that uncontrolled hypertension and uncontrolled diabetes mellitus (designated by AHA-defined “poor” status for blood pressure: SBP ≥140 mmHg or DBP ≥90 mmHg, and fasting blood glucose: ≥126 mg/dl) are conditions associated with a lack of health insurance coverage [30–32]. Prior data in a similar NHANES adult population demonstrate that uninsured hypertensive adults were more likely to be uncontrolled than their insured peers, and uninsured diabetics were twice as likely to be previously undiagnosed than insured diabetics, but these data do not explain how the lack of health insurance coverage impacts individual CV health factors’ contribution to overall ideal CV health [30, 32]. Analyses of health insurance coverage and chronic conditions typically show that adults without health insurance are less likely to be aware of personal diagnoses, less likely to receive screening for and achieve control of chronic medical conditions, such as hypercholesterolemia and hypertension [7, 8, 31–33] and less likely to receive preventive care including routine check-ups, hypertension screening, cholesterol screening, weight loss counseling, and smoking cessation [34]. This disparity may put uninsured individuals at increased risk of having undiagnosed or uncontrolled conditions, including hypertension, hypercholesterolemia, and diabetes mellitus. Observations in the present study suggest that by expanding health insurance coverage, cases of uncontrolled hypertension and uncontrolled diabetes mellitus may decrease, thus shifting the overall CV health of U.S. adults closer to ideal.

While a lack of health insurance coverage appears to be associated with uncontrolled hypertension and diabetes mellitus, our results show an inverse association between a lack of health insurance coverage and both obesity (designated by the AHA-defined poor status for BMI: BMI ≥ 30 kg/m^2^) and hypercholesterolemia. These findings may be attributed to an uninsured population in the United States that is disproportionately younger [35] and our inability to adjust for additional age-related confounders, including metabolic rate [36] that likely affect BMI and blood cholesterol. Our findings differ from previous studies in which uninsured individuals were observed to have a higher BMI and a higher prevalence of obesity than and similar or lower total blood cholesterol levels as their insured peers [33, 34, 37, 38]. For instance, in a study of a predominantly white adult population in the Framingham Heart Study, uninsured women had significantly higher mean BMI (28.0 kg/m^2^ versus 26.5 kg/m^2^; p = 0.02), a higher prevalence of obesity (34% versus 23%; p = 0.01), and comparable total blood cholesterol levels (193 ± 36 mg/dL versus 195 ± 40 mg/dL; p = 0.67) [33]. While our inability to adjust for additional confounders may explain some variation in findings, it is also possible that our results differed from previous studies due to differences in the composition of the study populations. NHANES data are representative of the general U.S. population and possess geographic variability and racial/ethnic diversity that most other population-based cohorts do not.

Consistent with our observations, other data suggest that there may, in fact, be no association between poor BMI levels and lack of health insurance coverage. For example, a 2010 state-by-state analysis of insurance coverage for obesity treatments found that if an obese patient obtained individual health insurance, there was no guarantee that obesity-related treatment would be covered under his or her health plan [39]. Furthermore, at the time the NHANES cycles analyzed in this study were conducted, states were not insuring recommended screening and treatment of adults for obesity through public or private insurance [39]. Further research is needed to see the impact on CV health of more recent insurance coverage for obesity and hypercholesterolemia screening, diagnosis, and treatment.

Regarding CV health behaviors, we observed an association between a lack of health insurance coverage and those with poor smoking status (current smokers), a finding consistent with prior studies [7, 28, 33, 37]. Reduced access to preventive care, particularly smoking cessation, may explain the poor smoking status of uninsured individuals, as insurance coverage of evidence-based smoking cessation treatments has been shown to lead to increases in quit attempts, use of cessation treatments, and successful smoking cessation [40]. Increasing health insurance coverage may be one way to expand the reach of cessation efforts; however, increasing coverage appears to be only one part of the solution [41]. For insured patients to take full advantage of cessation coverage, it should also be promoted within health plans adequately [41]. Smoking cessation appears to require a multi-faceted approach, and our findings show that expanding health insurance coverage may play a role.

Consistent with previous work [12], engaging in physical activity appears to be an additional CV health behavior that is linked to health insurance status, while dietary intake appears to be unrelated. Data from the National Health Interview Survey has shown that uninsured adults are more likely to be inactive than adults insured by private insurance, Medicaid or other forms of health insurance [42–45]. Though this association could be explained by overall SES, health insurance coverage may be a primary contributor, as lacking coverage is one marker of lower SES. Adults with a lower SES often experience greater barriers to exercise than their higher SES peers, particularly with respect to transportation and costs [46]. Health insurance coverage may aid in the reduction of costs for physical activity programs and facilities for individuals, thus alleviating one of the often cited barriers to physical activity by low SES adults.

Interventions that shift at-risk individuals, particularly those without health insurance, from poor toward ideal cardiovascular health are essential. During the first quarter of 2015, the uninsured rate among Americans dropped to 11.9%, its lowest point in seven years, with success largely credited to the roll out of the Affordable Care Act during the last quarter of 2013 [47]. Despite the steady improvement, U.S. adults continue to live without health insurance and are likely not receiving the preventive care and disease management necessary for ideal CV health. Our study suggests that interventions designed to target those CV health factors and behaviors associated with a lack of health insurance coverage (i.e. poor blood glucose control, poor blood pressure control, poor levels of PA, smoking) can potentially facilitate this shift towards ideal cardiovascular health for uninsured populations. To reach at-risk populations, community-based interventions may offer an effective population approach to target prevalent obesity and shift cardiovascular health towards ideal [48–50]. Community-based interventions can address disparities in cardiovascular health across racial/ethnic groups, particularly among those with limited access to care in clinical settings [51]. Uninsured adults are one sub-population with limited access to care in clinical settings, so expanding community-based interventions to reach this at-risk group may contribute to improved CV health and reduce health disparities.

Strengths of this study include the use of the National Health and Nutrition Examination Survey, a nationally representative sample, for our analysis, and the ability to gather data on all seven measures of CV health. However, limitations of this analysis must be acknowledged. We have documented an association between health insurance coverage and ideal cardiovascular health, but the possibility of unmeasured confounding requires further investigation. Our analysis focused specifically on health insurance coverage and did not take into consideration the usual source of care of survey respondents. In previous studies, those individuals (both uninsured and insured) who reported a regular, ongoing relationship with a health service facility or provider were more likely than those without a usual source of care to access health services [52, 53], suggesting that health care seeking behaviors are multi-factorial and not solely dependent on health insurance coverage. Additionally, out-of-pocket expenses (cost sharing) and setting of care are unknown. In the RAND Health Insurance Experiment, cost sharing reduced the use of nearly all health services, worsened blood pressure control, was associated with a decrease in use of essential medications and was associated with an increase in adverse events among the poor and elderly [54, 55]. Also, the cross-sectional design of NHANES, limited our ability to measure continuity of coverage, duration of insurance coverage and temporal changes in the health care system which would likely impact our findings, as irregular insurance coverage has been associated with poor health among middle-aged persons and the near-elderly [56, 57]. Lastly, although measurements are based on validated survey instruments, the CV health behaviors (e.g. PA, dietary intake, smoking status) are subject to self-reported measurement error.

Our results suggest that public policy initiatives that focus on improving access to health insurance may play a role in improving individual cardiovascular health factors and behaviors and overall cardiovascular health for the U.S. population. Additionally, community-level interventions targeting blood glucose and blood pressure control, smoking and physical inactivity may be most effective at improving CV health for uninsured populations. Future studies should aim to further examine the relationship between health insurance coverage and CV health and further explore community-based strategies for reaching this at-risk population in the United States.

## References

[1] Go AS, Mozaffarian D, Roger VL, Benjamin EJ, Berry JD, Blaha MJ, et al Heart disease and stroke statistics—2014 update: a report from the American Heart Association. Circulation. 2014;129(3):e28–e292. Epub 2013/12/20. 10.1161/01.cir.0000441139.02102.80 . 24352519 PMC5408159

[2] Ford ES, Greenlund KJ, Hong Y. Ideal cardiovascular health and mortality from all causes and diseases of the circulatory system among adults in the United States. Circulation. 2012;125(8):987–95. Epub 2012/02/01. 10.1161/circulationaha.111.049122 . 22291126 PMC4556343

[3] Dong C, Rundek T, Wright CB, Anwar Z, Elkind MS, Sacco RL. Ideal cardiovascular health predicts lower risks of myocardial infarction, stroke, and vascular death across whites, blacks, and hispanics: the northern Manhattan study. Circulation. 2012;125(24):2975–84. Epub 2012/05/24. 10.1161/circulationaha.111.081083 22619283 PMC3396556

[4] Yang Q, Cogswell ME, Flanders WD, Hong Y, Zhang Z, Loustalot F, et al Trends in cardiovascular health metrics and associations with all-cause and CVD mortality among US adults. JAMA. 2012;307(12):1273–83. Epub 2012/03/20. 10.1001/jama.2012.339 . 22427615 PMC9004324

[5] Lloyd-Jones DM, Hong Y, Labarthe D, Mozaffarian D, Appel LJ, Van Horn L, et al Defining and setting national goals for cardiovascular health promotion and disease reduction: the American Heart Association's strategic Impact Goal through 2020 and beyond. Circulation. 2010;121(4):586–613. Epub 2010/01/22. 10.1161/CIRCULATIONAHA.109.192703 . 20089546

[6] United States Department of Health and Human Services. About Healthy People 2010 [22 May 2014]. Available from: http://www.healthypeople.gov/2020/about/default.aspx.

[7] DeVoe JE, Fryer GE, Phillips R, L G. Receipt of preventive care among adults: insurance status and usual source of care. Am J Public Health. 2003;93(5):786–91. 12721145 10.2105/ajph.93.5.786 PMC1447840

[8] Davidoff A, Kenney G. Uninsured Americans with Chronic Health Conditions: Key Findings from the National Health Interview Survey. Urban Institute 2005.

[9] Smith J.C., Medalia C. Health Insurance Coverage in the United States: 2013 Washington, D.C.: U.S. Census Bureau, Current Population Reports; 2014 p. 60–250.

[10] Kontos EZ, Emmons KM, Puleo E, Viswanath K. Communication inequalities and public health implications of adult social networking site use in the United States. J Health Commun. 2010;15 Suppl 3:216–35. Epub 2010/12/22. 10.1080/10810730.2010.522689 21154095 PMC3073379

[11] Franks P, Clancy CM, Gold MR. Health insurance and mortality: Evidence from a national cohort. JAMA. 1993;270(6):737–41. 10.1001/jama.1993.03510060083037 8336376

[12] Wilper AP, Woolhandler S, Lasser KE, McCormick D, Bor DH, Himmelstein DU. Health Insurance and Mortality in US Adults. Am J Public Health. 2009;99(12):2289–95. 10.2105/ajph.2008.157685 19762659 PMC2775760

[13] Sommers BD, Long SK, Baicker K. Changes in Mortality After Massachusetts Health Care Reform: A Quasi-experimental Study. Annals of Internal Medicine. 2014;160(9):585–93. 10.7326/m13-2275 24798521

[14] Sloane DC, Diamant AL, Lewis LB, Yancey AK, Flynn G, Nascimento LM, et al Improving the nutritional resource environment for healthy living through community-based participatory research. J Gen Intern Med. 2003;18(7):568–75. Epub 2003/07/10. 12848840 10.1046/j.1525-1497.2003.21022.x PMC1494887

[15] Kirkpatrick SI, Dodd KW, Reedy J, Krebs-Smith SM. Income and race/ethnicity are associated with adherence to food-based dietary guidance among US adults and children. J Acad Nutr Diet. 2012;112(6):624–35. 22709767 10.1016/j.jand.2011.11.012 PMC3775640

[16] Pickering TG, Hall JE, Appel LJ, Falkner BE, Graves J et al Recommendations for blood pressure measurement in humans and experimental animals: Part 1: Blood pressure measurement in humans: a statement for professionals from the subcommittee of professional and public education of the American Heart Association Council on High Blood Pressure Research. Hypertension. 2005;45:142–61. 10.1161/01.hyp.0000150859.47929.8e 15611362

[17] United States Department of Agriculture, Food and Nutrient Database for Dietary Studies 4.1 (2010). Beltsville, MD: U.S. Department of Agriculture, Agricultural Research Service, Food Surveys Research Group.

[18] USDA, Agricultural Research Service. USDA Food and Nutrient Database for Dietary Studies, 5.0. Food Surveys Research Group Home Page, http://www.ars.usda.gov/ba/bhnrc/fsrg.

[19] Bowman SA, Clemens JC, Friday JE, Thoerig RC, Shimizu M, Barrows BR, et al Food Patterns Equivalents Database 2007–08: Methodology and User Guide: Food Surveys Research Group, Beltsville Human Nutrition Research Center, Agricultural Research Service, U.S. Department of Agriculture, Beltsville, Maryland; [17 3 2015]. Available from: http://www.ars.usda.gov/SP2UserFiles/Place/80400530/pdf/fped/FPED_0708.pdf.

[20] Bowman SA, Clemens JC, Thoerig RC, Friday JE, Shimizu M, Moshfegh AJ, et al Food Patterns Equivalents Database 2009–10: Methodology and User Guide: Food Surveys Research Group, Beltsville Human Nutrition Research Center, Agricultural Research Service, U.S. Department of Agriculture, Beltsville, Maryland .; [17 3 2015]. Available from: http://www.ars.usda.gov/ba/bhnrc/fsrg.

[21] Centers for Disease Control and Prevention (CDC). National Center for Health Statistics (NCHS). National Health and Nutrition Examination Survey Data. Hyattsville, MD: U.S. Department of Health and Human Services, Centers for Disease Control and Prevention, 2007 Available at: http://wwwn.cdc.gov/Nchs/Nhanes/Search/DataPage.aspx?Component=Dietary&CycleBeginYear=2007.

[22] Centers for Disease Control and Prevention (CDC). National Center for Health Statistics (NCHS). National Health and Nutrition Examination Survey Data. Hyattsville, MD: U.S. Department of Health and Human Services, Centers for Disease Control and Prevention, 2009 Available at: http://wwwn.cdc.gov/Nchs/Nhanes/Search/DataPage.aspx?Component=Dietary&CycleBeginYear=2009.

[23] Han E, Powell LM. Consumption patterns of sugar-sweetened beverages in the United States. Journal of the Academy of Nutrition and Dietetics. 2013;113(1):43–53. 10.1016/j.jand.2012.09.016 23260723 PMC3662243

[24] Wang YC, Bleich SN, Gortmaker SL. Increasing caloric contribution from sugar-sweetened beverages and 100% fruit juices among US children and adolescents, 1988–2004. Pediatrics. 2008;121(6):1604–14. 10.1542/peds.2007-2834 18519465

[25] US Department of Health and Human Services. Dietary Guidelines for Americans, 2005 6th ed. Washington, D.C.: US Government Printing Office.

[26] Appel LJ, Brands MW, Daniels SR, Karanja N, Elmer PJ, FM S. Dietary approaches to prevent and treat hypertension: a scientific statement from the American Heart Association. Hypertension. 2006;47:296–308. 16434724 10.1161/01.HYP.0000202568.01167.B6

[27] Johnson RK, Appel LJ, Brands M, Howard BV, Lefevre M, Lustig RH, et al on behalf of the American Heart Association Nutrition Committee of the Council on Nutrition, Physical Activity, and Metabolism and the Council on Epidemiology and Prevention: Dietary sugars intake and cardiovascular health: a scientific statement from the American Heart Association. Circulation. 2009;120(120):1011–20. 19704096 10.1161/CIRCULATIONAHA.109.192627

[28] McWilliams JM ZA, Meara E, Ayanian JZ. Health insurance coverage and mortality among the near-elderly. Health affairs (Project Hope). 2004;23:223–33. 15318584 10.1377/hlthaff.23.4.223

[29] Sorlie PD, Johnson NJ, Backlund E, Bradham DD. Mortality in the uninsured compared with that in persons with public and private health insurance. Arch Intern Med. 1994;154:2409–16. 7979836

[30] Wilper AP, Woolhandler S, Lasser KE, McCormick D, Bor DH, Himmelstein DU. Hypertension, diabetes, and elevated cholesterol among insured and uninsured U.S. adults. Health Aff (Millwood). 2009;28(6):w1151–9. Epub 2009/10/22. 10.1377/hlthaff.28.6.w1151 . 19843553

[31] Ayanian JZ, Zaslavsky AM, Weissman JS, Schneider EC, Ginsburg JA. Undiagnosed hypertension and hypercholesterolemia among uninsured and insured adults in the Third National Health and Nutrition Examination Survey. Am J Public Health. 2003;93(12):2051–4. Epub 2003/12/04. 14652333 10.2105/ajph.93.12.2051 PMC1448151

[32] Duru OK, Vargas RB, Kermah D, Pan D, Norris KC. Health insurance status and hypertension monitoring and control in the United States. American journal of hypertension. 2007;20(4):348–53. Epub 2007/03/28. 10.1016/j.amjhyper.2006.11.007 . 17386339

[33] Brooks EL, Preis SR, Hwang SJ, Murabito JM, Benjamin EJ, Kelly-Hayes M, et al Health insurance and cardiovascular disease risk factors. Am J Med. 2010;123(8):741–7. Epub 2010/07/31. 10.1016/j.amjmed.2010.02.013 ; PubMed Central PMCID: PMCPmc2913281. 20670729 PMC2913281

[34] Ayanian JZ, Weissman JS, Schneider EC, Ginsburg JA, Zaslavsky AM. Unmet health needs of uninsured adults in the United States. JAMA. 2000;284(16):2061–9. 10.1001/jama.284.16.2061 11042754

[35] DeNavas-Walt C, Proctor BD, Smith JC. Income and poverty in the United States: 2013: United States Census Bureau; 2014.

[36] Lazzer S, Bedogni G, Lafortuna CL, Marazzi N, Busti C, Galli R, et al Relationship between basal metabolic rate, gender, age, and body composition in 8,780 white obese subjects. Obesity (Silver Spring). 2010;18(1):71–8. Epub 2009/05/30. 10.1038/oby.2009.162 . 19478787

[37] Fowler-Brown A, Corbie-Smith G, Garrett J, Lurie N. Risk of Cardiovascular Events and Death—Does Insurance Matter? Journal of General Internal Medicine. 2007;22(4):502–7. 10.1007/s11606-007-0127-2 . 17372800 PMC1829431

[38] Schober SE, Makuc DM, Zhang C, Kennedy-Stephenson J, Burt V. Health insurance affects diagnosis and control of hypercholesterolemia and hypertension among adults aged 20–64: United States, 2005–2008. NCHS Data Brief. 2011;(57):1–8. Epub 2011/05/20. . 21592420

[39] Lee JS, Sheer JLO, Lopez N, Rosenbaum S. Coverage of Obesity Treatment: A State-by-State Analysis of Medicaid and State Insurance Laws. Public Health Reports. 2010;125(4):596–604. . 20597460 10.1177/003335491012500415 PMC2882611

[40] Treating tobacco use and dependence: 2008 update U.S. Public Health Service Clinical Practice Guideline executive summary. Respir Care. 2008;53(9):1217–22. Epub 2008/09/24. . 18807274

[41] Singleterry J, Jump Z, Lancet E, Babb S, MacNeil A, Zhang L. State medicaid coverage for tobacco cessation treatments and barriers to coverage—United States, 2008–2014. MMWR Morb Mortal Wkly Rep. 2014;63(12):264–9. Epub 2014/03/29. . 24670928 PMC5779348

[42] Pleis JR, Ward BW, Lucas JW. Summary health statistics for U.S. adults: National Health Interview Survey, 2009. Vital Health Stat 10 2010;(249):1–207. Epub 2011/09/13. . 21905346

[43] Schiller JS, Lucas JW, Ward BW, Peregoy JA. Summary health statistics for U.S. adults: National Health Interview Survey, 2010. Vital Health Stat 10 2012;(252):1–207. Epub 2012/07/28. . 22834228

[44] Schiller JS, Lucas JW, Peregoy JA. Summary health statistics for u.s. Adults: national health interview survey, 2011. Vital Health Stat 10 2012;(256):1–218. Epub 2012/12/01. . 25116400

[45] Blackwell DL, Lucas JW, Clarke TC. Summary health statistics for U.S. adults: national health interview survey, 2012. Vital Health Stat 10 2014;(260):1–161. Epub 2014/05/14. . 24819891

[46] Chinn DJ, White M, Harland J, Drinkwater C, Raybould S. Barriers to physical activity and socioeconomic position: implications for health promotion. J Epidemiol Community Health. 1999;53(3):191–2. Epub 1999/07/09. 10396499 10.1136/jech.53.3.191 PMC1756843

[47] Levy J. In U.S., Uninsured Rate Dips to 11.9% in First Quarter. Gallup-Healthways Well-Being Index [Internet]. April 20, 2015. Available from: http://www.gallup.com/poll/182348/uninsured-rate-dips-first-quarter.aspx?utm_source=Well-Being&utm_medium=newsfeed&utm_campaign=tiles.

[48] Rose G. Sick individuals and sick populations. Int J Epidemiol. 1985;14(1):32–8. Epub 1985/03/01. . 3872850 10.1093/ije/14.1.32

[49] Blackburn H. Population strategies of cardiovascular disease prevention: scientific base, rationale and public health implications. Ann Med. 1989;21(3):157–62. Epub 1989/06/01. . 2669850 10.3109/07853898909149926

[50] Mozaffarian D, Afshin A, Benowitz NL, Bittner V, Daniels SR, Franch HA, et al Population approaches to improve diet, physical activity, and smoking habits: a scientific statement from the American Heart Association. Circulation. 2012;126(12):1514–63. Epub 2012/08/22. 10.1161/CIR.0b013e318260a20b 22907934 PMC3881293

[51] Yancey AK, Kumanyika SK, Ponce NA, McCarthy WJ, Fielding JE, Leslie JP, et al Population-based interventions engaging communities of color in healthy eating and active living: a review. Prev Chronic Dis. 2004;1(1):A09 Epub 2005/01/07. 15634371 PMC544532

[52] Weissman JS, Stern R, Fielding SL, Epstein AM. Delayed access to health care: risk factors, reasons, and consequences. Ann Intern Med. 1991;114(4):325–31. Epub 1991/02/15. . 1899012 10.7326/0003-4819-114-4-325

[53] Gross CP, Mead LA, Ford DE, Klag MJ. Physician, heal Thyself? Regular source of care and use of preventive health services among physicians. Arch Intern Med. 2000;160(21):3209–14. Epub 2000/11/23. . 11088080 10.1001/archinte.160.21.3209

[54] Tamblyn R, Laprise R, Hanley JA, Abrahamowicz M, Scott S, Mayo N, et al Adverse events associated with prescription drug cost-sharing among poor and elderly persons. JAMA. 2001;285(4):421–9. Epub 2001/03/10. . 11242426 10.1001/jama.285.4.421

[55] Rea Brook. The Health Insurance Experiment: A Classic RAND Study Speaks to the Current Health Care Reform Debate. Santa Monica, CA: RAND Corporation, 2006 Available: http://www.rand.org/pubs/research_briefs/RB9174. Accessed: 21 April 2015.

[56] Baker DW, Sudano JJ, Durazo-Arvizu R, Feinglass J, Witt WP, Thompson J. Health insurance coverage and the risk of decline in overall health and death among the near elderly, 1992–2002. Med Care. 2006;44(3):277–82. Epub 2006/02/28. 10.1097/01.mlr.0000199696.41480.45 . 16501400

[57] Baker DW, Sudano JJ, Albert JM, Borawski EA, Dor A. Lack of health insurance and decline in overall health in late middle age. N Engl J Med. 2001;345(15):1106–12. Epub 2001/10/13. 10.1056/NEJMsa002887 . 11596591

